# Non-Market Values in a Cost-Benefit World: Evidence from a Choice Experiment

**DOI:** 10.1371/journal.pone.0165365

**Published:** 2016-10-26

**Authors:** Florian V. Eppink, Matthew Winden, Will C. C. Wright, Suzie Greenhalgh

**Affiliations:** 1 Landcare Research Manaaki Whenua, Auckland, New Zealand; 2 Institute for Biodiversity, Regensburg, Germany; 3 Department of Economics, University of Wisconsin, Whitewater, Wisconsin, United States of America; University of Waikato, NEW ZEALAND

## Abstract

In support of natural resource and ecosystem service policy, monetary value estimates are often presented to decision makers along with other types of information. There is some evidence that, presented with such ‘mixed’ information, people prioritise monetary over non-monetary information. We conduct a discrete choice experiment among New Zealand decision makers in which we manipulate the information presented to participants. We find that providing explicit monetary information strengthens the pursuit of economic benefits as well as the avoidance of environmental damage. Cultural impacts, of which we provided only qualitative descriptions, did not affect respondents’ choices. Our study provides further evidence that concerns regarding the use of monetary information in decisions with complex, multi-value impacts are valid. Further research is needed to validate our results and find ways to reduce any bias in monetary and non-market information.

## Introduction

Many researchers have expressed concerns about the increasing use of monetary value estimates in natural resources and ecosystem services policy and decisions [1, 2–4]. In practice, the use of monetary values in decision making should be decided on a case-by-case basis [5], but using a different basis for information can create different policy recommendations [6]. We contribute to the debate by presenting evidence that explicit monetary information affects choice outcomes when the full information set for a decision is ‘mixed’ and includes both monetary and non-market information.

There are several lines of criticism on estimating monetary values to inform natural resource and ecosystem service policy. McCauley [1] advises against the use of monetary information and market ethics because, under many conditions, they can also be used to justify environmental degradation. Gómez-Baggethun and Ruiz-Pérez [2] outline how ongoing valuation studies contribute to this possibility by (falsely) changing the perception of nature to a tradeable, substitutable commodity. Many have emphasised the technical difficulties of capturing the value of complex ecological systems that provide multiple and non-linear services in monetary units [3, 7]. Increasing attention is also being paid to the importance of cultural and heritage values [8, 9], which adds an additional dimension to policy development and decision making for which monetary value expressions may be difficult and inappropriate to estimate and use [10–12].

In practice, decision makers have to process and weigh various perspectives, interests and outcomes when deciding on conservation versus urban, infrastructural, rural or industrial development [13]. Monetary values provide easy comparisons between interests and are likely to remain in demand. If used with caution, monetary values can inform and contribute positively to natural resource and ecosystem service policy [5]. One of the questions Kallis et al. [5] propose to ask before using monetary values addresses what they call ‘complexity blinding’: is the use of monetary values likely to suppress other value articulations?

This question is pertinent to decision makers that regularly work with mixed information and provides ecosystem services research, a research field that could benefit from more structured goals and approaches [14, 15], with a testable hypothesis: in complex choices, monetary information is prioritised over other types of information. Although indirect evidence of this prioritisation exists in related motivation crowding studies [16], we found only a few studies that aim to identify the underlying cognitive process. The confirmation or rejection of this hypothesis would have important implications for the types of information collected during ecosystem service or natural resource assessments, and the way information is presented for management and policy decisions.

One experiment [17] that attempted to elicit how people interpret and use mixed information was conducted with 144 business economics students. In the experiment, the participants are asked to evaluate the performance of two hypothetical business units on the basis of a set of financial and other performance measures, such as customer orientation, and learning and growth opportunities. The measures where one business unit outperforms the other were manipulated, and the study suggested that differences in the financial measures were weighted more heavily than differences in other performance measures. Another experiment [18] presented 71 business economics students with information from an inclusive cost-benefit analysis (CBA) for a hydropower dam in Bolivia. Each group is split into treatments, with one group seeing economics-based information, one group receiving information that focuses on the importance of conservation, and a third receiving a combination of these types of information. The participants are then asked to rate their approval or opposition to building the hydropower dam in the presence and absence of trade-offs between the dam and conservation. Their results indicate that the students primed with economic material were more likely to prefer construction of the dam.

We conduct a discrete choice experiment among civil servants and democratically elected decision makers in New Zealand to determine the effect of monetary value information on the preferences of practitioners. We use an urban land use decision context for the choice experiment. This is an issue facing many decision makers around the world, including those in New Zealand. The case we use for the choice experiment reflects actual decision contexts facing urban areas in New Zealand.

In New Zealand, land use planning is to a large extent devolved to local decision makers, the Regional Councils and Territorial and Unitary Authorities. The central government describes what it wants policies to achieve, but leaves it to local decision makers to draw up land use plans and other policies to give effect to the central government’s guidance. Similar decision structures exist or are being formulated for other policy areas, such as transport and freshwater management.

The Resource Management Act 1991 (RMA) states that the environment should be sustainably managed and is a key document by which most plans and decisions with environmental impacts are judged. The RMA furthermore dictates that decision makers recognise the culture of Māori, the original inhabitants of New Zealand, and respect the principles of the Treaty of Waitangi, which recognised Māori ownership of their lands, forests and other properties while giving Britain sovereignty over New Zealand. In practice, the required consultation is not always effective, for instance because not all tribes have the resources to respond to the many requests for consultation. There are furthermore issues of trust relating to history, different world views and acceptable approaches to policy as well as research.

The remainder of the paper is structured as follows: first, we outline the experimental design; second, we describe the underlying theoretical framework and econometric methods employed; third, we present the empirical results; we conclude with a discussion of the policy implications of our results.

## Experimental Design

The goal of this study is to improve our understanding of the cognitive processing of monetary and non-monetary information by actual decision makers. Considering that decisions about development on the rural-urban boundary can involve complex, multi-value information, we framed the choice experiment in that context. The attributes of the choice experiment (development, water quality, and cultural heritage impacts) were initially selected based on our experience with projects conducted for local, regional and national government as well as other organisations in New Zealand that implement landscape-level projects.

We elected to conduct the online choice experiment with members of Regional and District Councils in New Zealand and the civil servants that prepare decision information. These groups make or are involved in development decisions on a regular basis. Understanding their choices in response to different information is of particular significance to sustainable development. To verify the salience of the attributes, we sent a draft survey to a number of retired decision makers. Furthermore, we tested the survey with a group of students. We initially presented the attributes and levels visually, but after testing replaced the visuals with text for greater balance between attributes. The experiment was vetted by Landcare Research’s Social Ethics committee for its accordance with the guidelines of the New Zealand-based Association of Social Science Research.

Respondents were notified of the survey through a formal letter, and subsequently invited to participate via email. Two follow-up emails were sent bi-weekly to respondents who had not completed the survey, and then on a weekly basis for another month. After the survey closed, we had 164 completed surveys (17.4% response rate), 75% of which had been completed by Regional and District Councillors. Table 1 shows that other respondent characteristics are distributed fairly evenly across the full sample.

**Table 1:** Sample means for various respondent characteristics.

	Share of respondents (%)
**Gender**	
Female	32.5
Male	67.5
**Age**	
<40	8.4
40–49	14.8
50–59	27.7
60–69	36.8
> 70	12.3
**Personal residence**	
Rural	41.8
Urban	58.2
**City inhabitants**	
> 50,000	36.7
15,001–50,000	30.9
5,000–15,000	21.3
< 5,000	11.2

The attributes of the choice experiment (shown in Table 2) present a scenario of rural-urban development impacts in New Zealand. The country has only seven cities with populations larger than 100,000 individuals and the largest city, Auckland, has 1.4 million inhabitants and is 3.6 times larger than the next-biggest city, Wellington. There are an additional 24 cities and towns with populations larger than 10,000 individuals, and the remaining urbanised areas can be as small as a few hundred inhabitants. The considerations of Councils with the largest cities may not be representative for other Councils and significant urban expansion is unlikely in small towns. Presenting a relatable scenario to all potential decision makers was essential for the experiment. To establish a common reference point for the respondents, we ask them to imagine a city of 20,000 inhabitants facing 4% population growth. The growth rate is a lower bound of urban growth rates for growing urban areas observed by Statistics New Zealand [19].

**Table 2:** Attributes and levels.^a^

Attribute	Levels	Definitions
**Development**	None	No appropriate areas of land can be rezoned for urban development
		No expansion in business and housing stock occurs
		Development must take place somewhere else in the district
		*No increase in revenues or costs*
	Limited	Half the urban growth is accommodated
		Residential expansion through low-density housing
		No expansion of commercial stock
		*(additional information in treatment 2 and 3*: *District revenue increases by $1*,*000*,*000)*
	Residential	All the urban growth is accommodated
		Residential expansion through medium-density housing
		No expansion of commercial stock
		*(additional information in treatment 2 and 3*: *District revenue increases by $2*,*000*,*000)*
	Residential and Commercial	All the urban growth is accommodated
		Residential expansion through medium-density housing
		Commercial expansion includes retail shops and 'super store' retail (e.g., home-improvement stores)
		*(additional information in treatment 2 and 3*: *District revenue increases by $3*,*000*,*000)*
**Water quality**	Low	Water is very murky
		No game fish, few native birds
		Not safe for boating, fishing or swimming
		*(additional information treatment 3*: *Losses in well-being valued at $500*,*000)*
	Medium	Water is slightly murky
		No game fish, but abundant native birds
		Safe for boating and fishing, but not swimming
		*(additional information treatment 3*: *Losses in well-being valued at $250*,*000)*
	High	Water is clear
		Abundant game fish and native birds
		Safe for boating, fishing and swimming
		*(additional information treatment 3*: *No losses in well-being)*
**Cultural**	None	Scenic views of the surrounding landscape are unaffected
		Urban edge is closer to The Stables tavern
		The Stables tavern remains a noticeable, solitary structure
	Stables	Scenic views of the surrounding landscape are not blocked and largely unaffected
		Urban development surrounds The Stables tavern. The tavern is no longer a noticeable, solitary structure
	Stables & landscape	Residences and commercial centre are clearly visible in the surrounding landscape
		Urban development surrounds The Stables tavern. The tavern is no longer a noticeable, solitary structure

^a^ Additional information in treatments shown in italics.

We phrase the attributes to reflect a set of variables that decision makers would take into account when deciding on development options on a rural-urban boundary. In the choice experiment, urban expansion could be ‘limited’ (accommodating half the projected growth), ‘residential’ (accommodating all of the projected population growth), and ‘commercial’ (which adds a commercial area with a super store to the residential attribute level).

Water quality is a major environmental policy issue in New Zealand. Urban expansion affects several water quality indicators, such as base flow, temperature, clarity, dissolved nitrogen and contaminants [20]. The levels of the water quality attribute were introduced using a water quality ladder, with ‘high’ water quality describing a stream with abundant game fish and birds, and clear water that is safe for boating, fishing and swimming. When water quality is of ‘medium’ or ‘low’ level, these qualities are diminished or absent.

The final attribute, impacts on cultural heritage, was selected because monetary estimates of cultural values are incomplete [21], not very common [22], increasingly seen as an important policy consideration [8, 9], and a prime example of the concerns about monetary value estimation [10–12]. We chose not to describe cultural heritage in terms of Māori culture or as a heritage asset on the New Zealand Heritage List. These contexts are culturally sensitive and associated with strict regulation which we expected to trigger specific responses from decision makers. Instead, we describe a heritage attribute with two levels: ‘Stables impact’, in which an old tavern that is well-known among the local communities is surrounded by new buildings, and a ‘Stables & landscape’ impact, in which the urban expansion surrounds the tavern and disturbs the scenic views of the environment. Most people in New Zealand would be familiar with such a situation, and both Māori and non-Māori New Zealanders can have strong associations with the landscape [23, 24].

A ‘choice scenario’ consisted of 3 possible expansion project options: two expansion alternatives described by the attributes above and a status quo option. The status quo option consisted of no development, high water quality and no impact to the cultural heritage site (Table 1). Inclusion of the “status quo” allowed individuals to have an “opt-out” or “no-change” option and was necessary to maintain unbiased parameter estimates [25].

To identify the impact of providing monetary or non-monetary information, we apply three treatments to the attributes and their levels. Table 2 shows treatment 1, or the control treatment, which contains only qualitative descriptions of the attribute levels. Treatment 2 provides monetary values (shown in italics in Table 2) for levels of development. The monetary values were based on Council revenues in 2014 [26]. In treatment 3, we also give monetary values for the levels of water quality (also shown in italics in Table 2). It was challenging to find appropriate estimates for the value of water quality. We chose to present monetary values for the attribute levels that we considered to be significant, but not overly large in comparison to the benefits from development. In the regression analysis, we use simple dummy coding for both attribute levels and treatments.

**Fig 1:**
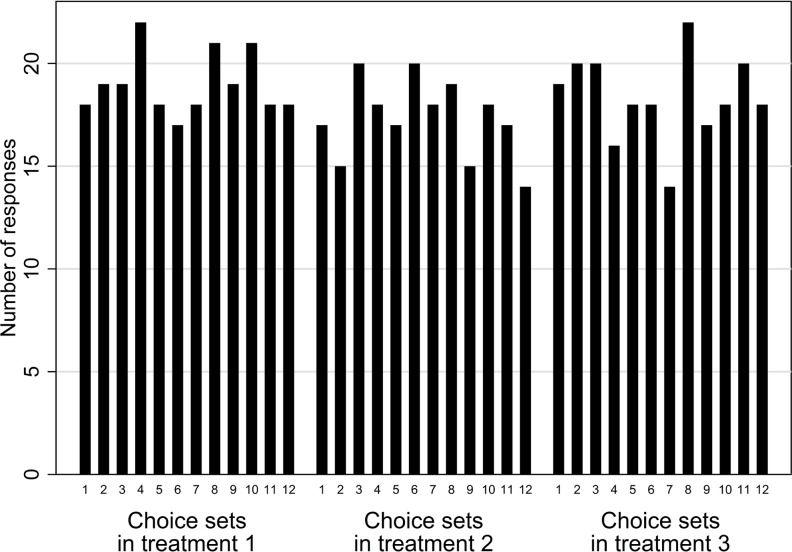
Responses per choice scenario.

We used Ngene software (v. 1.1.2; ChoiceMetrics) to generate a d-efficient fractional factorial design with 12 choice sets with two blocks of alternatives, each of which was accompanied by the option to retain the status quo. Respondents were randomly assigned to treatments upon accepting to conduct the survey, presented with the appropriate description of the attributes, and then were asked to complete four choice scenarios. Fig 1 shows this assignment procedure achieved an even distribution of responses across treatments and choice sets. Since each respondent completed four choice scenarios, a total of 656 observations were recoverable to estimate the preference relationship.

## Method

### Theoretical model

Analysis of individuals’ responses to the choice scenarios yielded the preference structure for the attributes. The presumption was the choice between development level, water quality and cultural heritage was driven by respondents’ underlying utility (either directly as an agent representing social interests, or because acting in the social interest maximises the likelihood of re-election which maximises the respondent’s private welfare). The utility function has two components (deterministic $\bar{V}$ (***x***_*ij*_, ***β***) and stochastic *ε*_*ij*_) and is therefore embedded in a random-utility framework denoted by (1) [27, 28].

$$U_{ij}=\bar{V}(x_{ij},\beta )+\varepsilon_{ij}$$ (1)

Subscript *i* denotes the individual, subscript *j* denotes the project, ***x*** is the vector of (1,2,…, *l*) attributes which vary across projects, and *ε*_*ij*_ is a stochastic error term capturing individual and alternative specific factors influencing utility unobservable by the researcher. The model was formalised by assuming the deterministic portion of utility can be approximated as a linear function of project attributes as represented by (2).

$$U_{ij}=\beta_{0}+\beta^{'}x_{ij}+\varepsilon_{ij}$$ (2)

Where *β*_*0*_ is a constant, and ***β'***_*ij*_ is the vector of *l* estimated coefficients for each attribute in ***x***_*ij*_. The probability, *P*_*ij*_, of a respondent, *i*, choosing a project, *j* or *k ≠ j*, is given by *U*_*ij*_ > *U*_*ik*_ as denoted in (3).

$$P_{ij}=\mathrm{Pr}(V_{ij}+\varepsilon_{ij}>V_{ik}+\varepsilon_{ik})=\mathrm{Pr}(\varepsilon_{ij}-\varepsilon_{ik}>V_{ik}-V_{ij})$$ (3)

### Econometric model

Statistical analysis of the choice experiment proceeded by estimating the utility difference model using both a conditional logit (CL) and mixed logit (ML) estimator. The mixed logit assumes stochastic variation in the preference structure, so that each individual has a unique *β_i_* for each of the attributes (overcoming the independence of irrelevant alternatives (IIA) property of the CL). These parameters are distributed in accordance with certain conditions in the population. The generalised extreme value distribution was assumed to be the probability distribution of the error term. When f(*β*|*θ*) is the density function for a given distribution of *β* with parameter *θ* (i.e. means and distributions), the choice probability can be expressed as (4).

$$Pij=\int \frac{\exp V_{ij}}{\exp V_{ik}}(\beta )\;f(\beta |\theta )\;d\beta$$ (4)

The normal distribution was assumed for f(*β*). The selection probability becomes P^*^ using simulated maximum likelihood procedures. *R* represents the number of draws from the density function with *β*^*r*^ representing the *r*^th^ draw. This resulted in a simulated probability, P^*^_ij_, denoted by (5).

$$P_{ij}^{*}=\frac{1}{R}\sum_{r}P_{ij}(\beta^{r})$$ (5)

P^*^_ij_ was used to estimate the simulated log likelihood function (LL^*^) and the parameter *θ* which defines the distribution that maximises this function. Given *d*_*ij*_ is a dummy variable representing respondents’ choices (1 when project *j* is chosen, 0 otherwise), LL* was estimated as denoted in (6).

$$LL^{*}=\sum_{i}\sum_{j}d_{ij}\ln(P_{ij}^{*})$$ (6)

The distribution function quantifying the variability in preferences found between respondents can be derived from these calculations [28].

All of the attributes comprising an urban-rural expansion project have well-defined expectations with respect to sign. Increases in development are expected to be preferred, while decreases in water quality and cultural heritage are not preferred. A common linear additive utility function is assumed for all respondents and allowed for estimation of society’s preference structure.

## Results

We estimated the effects of presenting monetary information on preferences of the full sample and a subsample of responses from Councillors using a CL model (models 1 and 2 in Table 3). Since we could not confirm the assumption of the independence of irrelevant alternatives for these models, we also estimated ML models with 500 shuffled Halton draws (models 3 and 4 in Table 3). The log likelihood scores of the four models indicate that the models for the Councillor sub-sample are improvements over the full sample of completed responses.

**Table 3:** Regression results.^a^

	(1)	(2)	(3)	(4)
	CL	CL	ML	ML
	All	Councillors	All	Councillors
**Main effects**				
**Development**				
Limited	4.260***	4.114***	6.819***	6.878***
	(0.000)	(0.000)	(0.002)	(0.011)
Residential	4.553***	4.545***	6.259***	6.554***
	(0.000)	(0.000)	(0.006)	(0.013)
Commercial	4.112***	4.184***	6.387***	6.663***
	(0.000)	(0.000)	(0.002)	(0.005)
**Water quality**				
Medium	-3.954***	-0.393***	-7.324***	-7.578***
	(0.000)	(0.000)	(0.000)	(0.000)
Low	-6.135***	-0.602***	-14.950***	-19.020***
	(0.000)	(0.000)	(0.000)	(0.001)
**Cultural**				
Stables	-0.822*	-0.758	-1.239	-2.131
	(0.080)	(0.150)	(0.294)	(0.244)
Stables & landscape	-1.672**	-1.850***	-2.765*	-2.366
	(0.031)	(0.001)	(0.080)	(0.164)
**Treatment effects**				
**Development**				
Limited	0.205	0.084	1.290	2.650
(monetary information shown)	(0.452)	(0.790)	(0.187)	(0.109)
Residential	0.670**	0.647*	3.222**	3.450**
(monetary information shown)	(0.018)	(0.058)	(0.015)	(0.062)
Commercial	0.308	0.254	1.754*	2.225
(monetary information shown)	(0.282)	(0.433)	(0.082)	(0.166)
**Water quality**				
Medium	-0.473*	-0.299	-2.272*	-2.085
(monetary information shown)	(0.076)	(0.323)	(0.064)	(0.135)
Low	-0.919*	-0.592	-4.305**	-8.649**
(monetary information shown)	(0.082)	(0.286)	(0.018)	(0.015)
Log likelihood	-852.9	-647.0	-421.92	-316.4
*N*	1,968	1,476	1,968	1,476

^a^ *p*-values are shown in parentheses.

*** indicates *p <* 0.01

** indicates *p <* 0.05, and

* indicates *p <* 0.1

The coefficients have the expected signs in all models. Some degree of development is always preferred over no development. The coefficients for medium and low water quality are negative relative to the base level of high water quality. In every model, the coefficient for low water quality is a multiple of the coefficient for medium water quality. This suggests that decision makers are willing to accept a limited reduction in water quality, but prefer to avoid severe reductions in water quality.

The coefficients for cultural heritage impacts are also negative with respect to the base level of no impact, indicating that there is a preference to avoid such impacts. The effect is not consistently significant, but more so when the landscape rather than a heritage building is affected by new urban development. We suspect that this result is a consequence of design choice; attribute levels framed as regulatory requirements or Māori culture may well have triggered a stronger preference for the conservation of cultural heritage.

The bottom half of Table 3 shows the treatment effects of presenting monetary information on decision makers’ preferences. We model the treatment effects as an interaction term between attribute levels and a dummy for whether monetary information was given for that attribute (1) or not (0). The coefficients of the interaction terms indicate respondents’ additional preference for a choice set when monetary information about its attribute levels is given.

The signs of the coefficients of the treatment effects correspond to our expectations: being shown monetary information about development tends to emphasise preferences for each development level, and monetary information about water quality causes a stronger aversion to water quality degradation. The effect appears to be stronger for water quality than for development. This suggests that providing monetary estimates of environmental impacts does make it more likely that such effects are taken into account by decision makers.

The treatment effect is significant in more instances in the ML model than in the CL model. For limited and residential development options, the treatment effect is significant or very close to significance. For urban expansion with commercial stock, the effect is less strongly significant. It may be that this represents an extreme development option for which it matters less whether monetary information is shown. For choices made regarding water quality, we see that the preference to avoid extreme water quality degradation is clearly strengthened by being shown monetary information. For an intermediate reduction in water quality, the effect is less strongly significant. This may be caused by an understanding that some loss in water quality is unavoidable, or by an expectation that additional tax revenues can be used to clean up or offset a decline in water quality.

**Fig 2:**
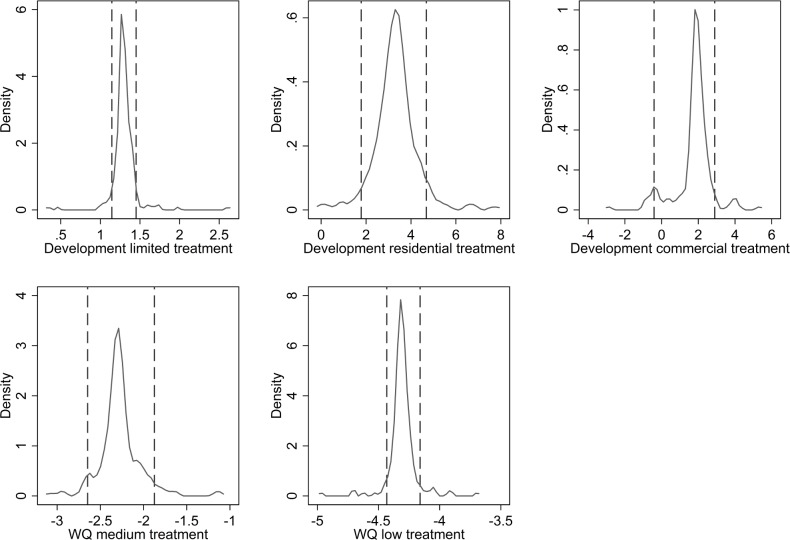
Density plots of the mean coefficients of the treatment effects. Plot**s** are for each respondent after 500 simulations. Vertical dotted lines indicate a 95^th^ percentile interval.

In each Halton draw, 15 individuals are randomly dropped from the sample, meaning there are on average 460 estimates of each coefficient for each respondent. Fig 2 shows density plots of the mean estimated coefficients of the treatment effects for individual respondents in model 4. Fig 2 is an aggregated visualisation of the mixed logit results, and the peaks of the plots correspond closely to the coefficients shown in Table 3. The left and right-hand vertical lines indicate the 95^th^ percentile-interval of respondents around that coefficient estimate; coefficient values outside this interval can be assumed to come from non-representative respondents.

The plots in Fig 2 lend further support for the conclusion that providing monetary information influences decision makers’ preferences. With the exception of preferences for the highest level of urban expansion, 95% of respondents who were shown monetary information have a stronger tendency towards a given attribute level than those who do not see monetary information for the same attribute level. The policy implication is that presenting decision makers with monetary values for only some of the variables in a complex decision makes it likely that they will focus on the variables with monetary values.

## Discussion

The growing use of monetary estimates for natural resources and ecosystem services to inform policy is increasingly seen as potentially problematic. There are various reasons for this concern, and we considered the possibility that monetary information tends to be prioritised over other types of information. We conducted a discrete choice experiment among decision makers in New Zealand to investigate the effect of monetary information on choice outcomes.

The results of the choice experiment provide strong evidence that monetary information strengthens preferences. When an increase in tax revenue is explicitly shown, the preference for any given development level is reinforced over choices made without that monetary information. Similarly, decision makers showed a tendency to avoid degradation of water quality, which was reinforced when they were given the monetary estimates of the associated welfare losses. By assessing the behaviour of individual respondents, it was shown that respondents’ choices were clearly and consistently affected by being shown monetary information.

The first policy implication is that monetary valuation of non-market benefits provides information that more strongly supports conservation of natural resources and ecosystem services. When such benefits are not monetised, the economic benefits of development, which are normally expressed in monetary units, are emphasised and likely to dominate decision makers’ choices.

A second policy implication is that all non-market impacts of a decision should be monetised to ensure decision makers weigh all impacts equally. This is not always possible, however, for practical or moral reasons. Cultural and heritage values are clear examples of impacts for which monetary valuation may be difficult and inappropriate

Our results indicate that the (mostly philosophical) concern about the use of monetary information for policy support is justified. Further work will be required, however, to understand the cognitive processing of mixed information. In order to determine if our results hold in general, similar studies need to be conducted in other contexts, with various groups of respondents and experimental designs. We used a simplified example of the information we would expect decision makers to see in real life. This may have introduced confounding statements in our effort to minimise the time the Councillors would need to complete the survey.

An improved, more realistic experimental design would further the insights into the cognitive processing of complex, multi-value decisions. Other elements of such decisions that could be considered are irreversible change, cumulative losses and uncertainty about impacts and benefits. The fact that some types of environmental damage can be repaired retroactively might also be relevant to decision makers’ choices.

Our study does not, however, present a solution to the finding that monetary information strengthens preferences and may lead to overly high losses in resources with non-market values that are not monetised. It may be informative to explore the formation of utility functions in the context of complex, mixed information decisions. Questions that emerge from that line of inquiry include if well-formed preferences exist in such decision contexts, and which methods help align choice outcomes more closely with a true utility function? There is a significant body of research on experimental design that can be drawn from to test ideas about how decisions affecting natural and cultural resources are made. We believe that such rigorously designed studies will strengthen applied policy research.

## Supporting Information

S1 Table Choice experiment responses. This file contains the data ready for use with Stata.
(DTA)
